# Perceptions of Art Therapy in Adolescent Clients Treated Within the School System

**DOI:** 10.3389/fpsyg.2020.518304

**Published:** 2020-11-09

**Authors:** Shir Harpazi, Dafna Regev, Sharon Snir, Racheli Raubach-Kaspy

**Affiliations:** ^1^Faculty of Social Welfare and Health Science, School of Creative Art Therapies, University of Haifa, Haifa, Israel; ^2^Department of Art Therapy, Faculty of Social Science & Humanities, Tel-Hai Academic College, Tel-Hai, Israel

**Keywords:** art therapy, education system, clients’ perceptions, adolescent, school-based

## Abstract

Research in School-Based Art Therapy has been widely discussed in recent years, and the number of studies that examine staff perceptions and the special characteristics of art therapy within the education system has risen considerably. The current study explored the critical issue of adolescent clients’ perceptions of art therapy in school, from their point of view as clients. The methodology and data analysis were conducted according to the principles of Consensual Qualitative Research (CQR). The sample was composed of 12 adolescent clients, aged 14–18 (*M* = 16), who took part in in-depth semi-structured interviews. The findings were organized into five domains that emerged from the interviews: referrals and initial engagement with therapy, the setting within school, the nature of art therapy at school, the relationship with the art therapist, and the impact of art therapy on these clients. The analysis revealed that although some participants initially agreed to art therapy because it got them out of class and let them have fun instead, they realized after a period of time of art therapy that they were engaged in a personal and emotional process focusing on them which allowed them to express their feelings without the fear of judgment. Participants at times used the word “mother” to describe their relationship with the art therapist, and stated that the presence of the art therapist at school made them feel safer and helped them deal with day-to-day problems. School-based art therapy was seen as having specific advantages according to the participants. Having a therapeutic hour during a stressful school day was considered to give these students an opportunity to relax, and the art therapy room was perceived as a shelter. In addition, when the therapist was perceived as a supportive figure, the whole school experience tended to be perceived as supportive or enabling greater acceptance.

## Introduction

In recent years, increasing numbers of art therapists have been incorporated into the school system as part of the rise in therapeutic services offered by the education system, as well as the growth in interest and research on the school-based art therapy around the world (Randick and Dermer, 2013; Snir et al., 2018; McDonald et al., 2019b). Art therapy in school provides a broad support system for the client, generates an opportunity for collaboration between the various actors in the child’s life, and unlike work in private clinics, and allows therapists to engage in teamwork with teachers and staff, which helps alleviate their sense of isolation (Regev et al., 2015; Snir et al., 2018). A recently conducted systematic review showed that the art therapy may be beneficial for children and adolescents with post-traumatic symptoms, delinquent behavior, and for those who have not been diagnosed with specific difficulties but are faced with other life challenges (Cohen-Yatziv and Regev, 2019).

Studies have reported strong associations between students’ emotional state and their academic and social achievements in school (Paternite, 2005; Suldo et al., 2014; Gryglewicz et al., 2018). In addition, the school climate and environment have an impact on the emergence of emotional, personal, and behavioral problems among adolescents (Fisher and Brown, 2018; Ogden and Hagen, 2018). For example, being involved in bullying incidents, either as an attacker or as a victim, or being socially rejected at school, may increase the risk of self-harm in adolescents (Esposito et al., 2019). By contrast, a more extensive therapeutic staff available within the school may contribute to students’ sense of security, foster their academic achievement, and decrease violence at school (Mann et al., 2019). Effective involvement of the school system, including support from the educational staffs and parents, can contribute to the reductions in risk behaviors (Wang and Fredricks, 2014). This suggests that school may constitute an important place for identifying children and youth at risk, or those facing difficulties, and may be a setting that can provide them with appropriate early treatment (Dunne et al., 2017).

A preliminary review of studies of primary-school-based art therapy indicate that art therapy in school may contribute to improvement in classroom behavior and can help reduce resistance and anxiety disorder symptoms (McDonald and Drey, 2018). In addition, the ability to exercise choice, the availability of time to oneself, and the opportunity to create and share, were perceived by children in this study as beneficial elements in their school experience. These children reported that art therapy sessions helped them to calm down and contributed to improvement in behavior, learning, and concentration levels. Despite these findings, the children’s perceptions of the reasons for their referral to therapy showed a lack of clarity and understanding. There were also disparities between the rationale for referral put forward by these children and the one presented by their teachers. This may be due to the fact that age, developmental level and children’s reflective ability make it difficult for them to observe and report on the complexities emerging from school-based art therapy, in addition to the role of the relationship with the therapist (Deboys et al., 2017; McDonald et al., 2019a).

Despite the acknowledgment of their rights as early as the 20th century, adolescents have only recently been perceived as a population with specific needs and characteristics (Dunne et al., 2017). Their specific needs are associated with the many physical and cognitive changes that occur during adolescence, but are also related to pressures and demands from the environment (Gatta et al., 2014; Ogden and Hagen, 2018). The difficulty of coping with maturation can manifest in behavioral disorders, mood disorders, depression, anxiety, or even lead to suicidal tendencies. This underscores the importance of developing appropriate responses to adolescents’ needs, since lack of support can negatively influence their development of social and behavioral competencies (Dunne et al., 2017; Gryglewicz et al., 2018).

Qualitative studies have examined the adolescents’ perceptions of psychotherapy, to better understand their specific perspective as clients. The findings indicate that adolescents are concerned about the therapist’s criticism, but also want the therapist to reveal themselves to some extent (Eyrich-Garg, 2008). Research has shown that adolescents value mutual respect and equality in their relationship with the therapist, and crave their therapist’s attention (Everall and Paulson, 2002; Gibson et al., 2016).

Understanding clients’ perceptions of the therapeutic process, therapists’ interventions, and their willingness to change, are all crucial factors in enabling the process of change (Levitt et al., 2016). Thus, a better understanding of clients’ specific constellations of traits and attitudes toward therapy, and their grasp of the benefits of the therapeutic process can help therapists provide an appropriate therapeutic response (Levitt et al., 2016; Noyce and Simpson, 2018). The present study explored how adolescents experience and perceive art therapy they receive at school, within the inherent complexity of the school setting. Specifically, it aimed to define the essential factors contributing to the therapy, from the point of view of adolescent clients.

## Materials and Methods

The methodology and data analysis were conducted according to the principles of Consensual Qualitative Research (CQR), which are based on phenomenological perception, and are designed to understand participants’ internal subjective experiences in-depth by integrating positivistic ideas (Hill, 2015). In the CQR method, the interviews are examined by at least three judges until agreement is reached on domains and core ideas emerging from them, thus enabling the emergence of different points of view that enhances the quality of data analysis. The researchers apply their clinical skills to comprehend and make sense of the data. At the same time, they are cognizant that this type of study does not generate an objective ground truth but rather constitutes an attempt to reach a consensus with regard to a subjective experience (Hill et al., 2005; Hill, 2015).

### Participants

The sample was composed of 12 adolescent clients, aged 14–18 (*M* = 16), eight girls and four boys. They had all taken part in art therapy sessions in a school setting for more than a year, individually or in groups. All the participants had relatively high verbal abilities, which were required since the primary mode of data collection in the present study was interviews.

The participants were enrolled in six different schools, from different areas of Israel, and were being treated by eight different art therapists. Three participants were in regular educational settings, five were in a regular educational school for at-risk youth, two were in special education classes within a regular school framework, and two were studying in a special education school.

Five of the participants were in individual therapy, five in group therapy, and two participants received both individual and group therapies during the year of the interview (Table 1).

**Table 1:** Demographics.

#	Gender	Grade	Educational setting	Time in Therapy	
1	Male	Twelfth grade	Special education class, integrated into the regular education setting	Third consecutive year in therapy	Individual
2	Female	Twelfth grade	Regular education, setting for at-risk youth	Third consecutive year in therapy	Group
3	Male	Twelfth grade	Regular school, for at-risk youth	Third consecutive year in therapy, two first years in a group, and current year individually	Individual
4	Male	Eleventh grade	Regular school, for at-risk youth	Second consecutive year in therapy	Group
5	Female	Eighth grade	Regular education setting	Third consecutive year in therapy	Group
6	Female	Eighth grade	Regular education setting	Third consecutive year in therapy	Group
7	Female	Eleventh grade	Regular school, for at-risk youth	Second consecutive year in therapy, the first year in group therapy, and the current year in both group and individual therapy	Both individual and group
8	Female	Eleventh grade	Regular school for at-risk youth	Second consecutive year in therapy, the first year in group therapy, and current year in individual therapy	Individual
9	Female	Eleventh grade	Special education school	Second consecutive year in therapy	Both individual and group
10	Male	Eighth grade	Regular education school	Second consecutive year in therapy	Group
11	Female	Eighth grade	Special education class, integrated into a regular school	Second consecutive year in therapy, the first year in group therapy, and the current year in individual therapy	Individual
12	Female	Eleventh grade	Special education school	Second consecutive year in therapy	Individual

### Tools

#### Semi-Structured Interviews

According to the CQR method, the primary research tool is a semi-structured, in-depth interview (Hill, 2015), which provides a means to explore the participants’ subjective experience (Creswell, 2014). The interviews were based on open-ended questions, which gave the interviewees great latitude to construct their answers in a way that seemed meaningful to them.

The interviews dealt with the interviewees’ experience of art therapy at school, their perceptions of art therapy (e.g., “tell me what art therapy in school means to you”), the therapeutic setting and the relationship with the therapist (e.g., “tell me about your relationship with the art therapist, who is she for you”), the influence of art therapy on the student’s entire school experience (e.g., “has being in art therapy affected your relationships with your classmates or with the educational staff?”), and the influence of the school setting on the experience and therapeutic process (e.g., “how does the art therapy integrate into the school routine?”). Then, as a function of the participant’s responses, more detailed questions were asked to further explore the topics mentioned, or not yet addressed, by the interviewee (Hill, 2015). As part of the data collection and analysis process, and to refine the research tool, adjustments were made to the interview guide as the interviews progressed.

#### The Researcher as the Research Tool

In qualitative-constructivist research, the researcher constitutes the research tool and forms an integral part of the study. However, this involvement raises methodological issues that require the researcher to have a clear-cut position, and work toward separating this position from the situation being studied, to be able to rethink the meanings of the experience (Creswell, 2014). Furthermore, the background and experience of the researcher are important components of the researcher as research tool (Shenton, 2004). Note that here, that the students did not know the first author, who was the main researcher and interviewer in the study. However, as a result of her years of working with adolescents in the education system as part of the educational staff, she felt a connection to this population.

### Procedure

To recruit the participants, we reached out to art therapists, directors of therapeutic centers, school administrators, and educational consultants. These individuals located potential students who met the inclusion criteria. All interviews took place in the schools where the students were enrolled. Each interview lasted from 20 min to 1 h. At the beginning of the interview, participants were told that they could stop the interview at any time, and that their identity would be kept confidential. As a part of the informed consent, the participants and their parents authorized the recording and transcription of the interviews.

### Data Analysis

As stipulated in the QCR data analysis method (Hill, 2015), three of the authors conducted the data processing and analysis in three stages. The first author of this article was, at the time of this study, a graduate student in art therapy. The second and third authors are art therapists and experienced researchers in the field. In the first stage, based on three interviews, each author analyzed the transcripts to extract the main domains arising from the data. Then, we met to discuss and reach a consensus on these domains. In the next stage, each author extracted the core ideas from the interviews, after which we met again to reach a consensus about core ideas. The last phase was carried out by the first author. This consisted of analyzing all the interviews to extract the domains and core ideas defined in the consensus stage.

In the description of the findings, the phrase “most participants” refers to over 75% of the interviews, the phrase “some participants” refers to 25–75% of the interviews, and the phrase “a few participants” refers to less than 25% of the cases (Hill, 2015). If an issue appeared more relevant to special or regular education students, it was noted as a specific related core idea.

### Ethical Considerations

Recruitment for this study was complex. There were cases where the therapists or the directors of therapy centers were concerned that the client’s identity will be inadvertently disclosed or that the study would impact therapy. Once collaboration was obtained, the art therapist forwarded the researcher’s contact and information to the parents. After obtaining parental permission, the study was presented to the adolescents, and if they also agreed to be interviewed, an interview was scheduled. In the explanatory letter addressed to the adolescents and their parents, it was made clear that participation was voluntary and that they could end the interview at any time without repercussions. They were also told that their responses were confidential and that all shared information would be kept anonymous, so that their identity would not be revealed at any stage of the study or in publications. In addition, the clients were told that the information provided in the interviews would not be passed on to the educational or therapeutic staff at the school, or their family, and would only be used for research purposes. The study was approved by the Chief Scientist at the Ministry of Education, and by the Ethics Committee of the Faculty of Health and Welfare at the University of Haifa.

## Findings

### Referral and Initial Engagement in Art Therapy in the Education System

#### The Referral Process and the Presentation of Art Therapy to the Clients

Most of the participants noted that they were referred to art therapy by the school’s educational staff, mainly educators, and counselors. The way therapy was mediated to the clients appears to have varied and at times, the presentation was either incomplete or unclear, and needed to be reformulated for each client individually. Some participants stated that they knew they were being referred to therapy to treat their emotional, personal, or classwork-related-stress issues, such as for example, their drive to excel: “because they think I study too much and that I need to stop from time to time and see that… I don’t always have to get to the top, I am too stressed.”

A few participants stated that therapy was presented as an art lesson or as an hour of talking or fun, without any mention of psychotherapy: “They said let’s arrange it for you once a week, you’ll have your down time, free time when you can do whatever you want, do art, talk, you can also sit and not do anything….” Some participants commented that this way of presenting the art therapy as a non-study hour instead of being in class convinced them to go to therapy: “At first it was more about avoiding class.” A few participants, all of them are students in special education frameworks, said that they knew in advance that they would be getting art therapy as a part of their school schedule: “It goes along with the classroom activities. Even in the last school, it was like that.”

#### Therapy Expectations and Goals

Some of the participants indicated that they did not have clear expectations, but came with an open mind, and were willing to see whether therapy was right for them. A few talked about their negative expectations and fears at the beginning of the art therapy sessions, which were associated with their uncertainty about privacy or lack of rapport with the therapist: “I thought, what is this BS? I don’t need it; I can handle things on my own. I was very closed off and didn’t trust anybody.” Some of the participants said that they expected the therapy to involve art-making alone, or only talking, and did not expect to develop a close relationship with the therapist.

Some of the participants were able to specify the initial goals they set for themselves in therapy, whereas others struggled to describe these goals throughout the entire interview. Some of the goals were associated with social issues, such as their wish to “be better socially,” or improve their communication skills with peers and family. The goals associated with the school context were associated with the school system’s ability to support them. The personal goals were related to having more self-confidence and greater self-expression, acceptance, and self-esteem: “I wanted to make myself more open… I wanted to present myself in other ways and be more confident. To be able to talk about my feelings without anyone judging me.”

### Therapeutic Setting in the School System

#### The Time and the Space for Art Therapy

This core idea deals with the technical features involved in providing art therapy in an educational setting. Two key factors emerged: how to fit art therapy into the school schedule and the physical setting of the art therapy room.

#### Fitting Art Therapy Into the School Schedule

All the participants pointed out that the art therapy hour is a permanent feature in the school schedule, takes place once a week, and is an integral part of their weekly routine at school. A few participants noted that therapy was scheduled during their free lesson, or at the end of the day, so that they did not get a break or could not go home early: “My session, it’s a little bit of a bummer because normally I could go home earlier, but never mind….” A few others noted that their therapy time was scheduled during another class hour and that they had to catch up on the lesson. Most of the participants indicated that they did not feel that therapy was negatively influenced by the holidays and other discontinuities related to the school calendar.

#### The Physical Setting of the Art Therapy Room

All the participants were satisfied with the art therapy room and with the equipment. Most noted that the room was appropriate, fulfilled their needs and sometimes let them disconnect from the school routine: “The room is huge, with a huge table and chairs, and we just sit in a corner, and I feel like we are in a world of our own.” A few noted that if some of the art materials had run out, they did not feel it was a problem, but could manage without them or “contributed” by bringing materials from home: “I don’t feel anything is missing, but if there is, me and (the therapist) bring our own stuff, she brings, I bring… we contribute to the room.”

Most of the participants indicated that the room was secluded and allowed for privacy. A few participants mentioned that sometimes there were interruptions when other students created noise by the room, but that they feel protected by the therapist.

#### Confidentiality of Therapy

All the participants noted that they felt comfortable about being in art therapy at school. A few said that although they felt comfortable, they would prefer not to call it “therapy.”

Most participants indicated that they did not attach much importance to whether students in their class knew they were in therapy: “I don’t know, I don’t care. I don’t care if they find out, I don’t care if they don’t’. It doesn’t matter to me.” A few participants even described the art therapy hour as a source of pride as compared to other students at school: “I even make fun of them because they don’t have this hour and I do. Everyone was dying to do it too.” On the other hand, a few participants, all enrolled in special education settings, said that they were happy knowing that all the students in their school get therapy, since they had concerns over stigma: “You do not feel different. Because if a normal school takes only certain kids, then it’s like, ‘He’s weird, he is not okay if he’s having it’… But here everyone, there’s no difference between students here.”

All the participants stated that they felt their privacy was protected and could count on the therapist and the rest of the staff to preserve confidentiality.

#### The Composition of Group Art Therapy in School

Some participants discussed the fact that the groups tended to fluctuate from year to year. Occasionally, this was due to a scheduling constraint or participants leaving the group, but other times changes were made to adapt the therapy to the needs of the clients or to schedule individual therapy for some of the former group members.

Some of the participants in group art therapy noted that the continuity of the group was crucial from year to year because it provided a sense of unity and intimacy in the group: “It is very significant; I think if someone is in the group, he/she should stay there until the end.” Some participants stated that they had problems in trusting other participants who were in therapy. All the participants who were familiar with both individual and group therapies emphasized that they felt more comfortable sharing personal content in individual therapy: “being in a group means less talking, because it’s a group, and there are things you don’t want anyone to know, and alone I don’t have a problem talking.”

### The Nature of Art Therapy Sessions in School

#### Feelings on Therapy Day

All the participants described the therapeutic hour as a break from the school routine: “An hour to relax. Take a break from all the schoolwork… you can barely breathe, it’s like, too much, like, you know, choking.” A few said that they viewed the art therapy as a gift or a privilege that they got from the school: “It’s an experience, it’s a gift. Not everyone deserves it.”

Most participants said that they looked forward to their therapy day and felt more willing about going to school at that day than during the rest of the week. A few participants noted that their feelings about therapy depended on their general feelings at school that day, and on busy or stressful days, they felt less like going to therapy: “It depends on the day. If there is pressure that day, then less. There are days when there is a lot of pressure from exams, finals, which make it more difficult (to find time for therapy).” A few participants also indicated that when they had problems or were angry with the school system as a whole, this could affect their perception of therapy as part of the school system. They tended to express this perception when they said “they” by referring to school related factors, including the art therapist as a single unit.

#### Therapy Process and Intervention Techniques

All the participants stated that they felt they were able to make choices during therapy and that they were given the opportunity to take the lead in determining how the session would unfold. Most participants said that the therapist allowed them to decide what they wanted to do in the session: “I choose. I come in, and she asks me ‘do you want to do something in art, choose some markers, watercolors’… or talk, sit with me and talk.” Most participants indicated that the sessions included both conversation and art making, depending on what they wanted. Sometimes the therapy could start with a conversation and then move onto art, while in other cases, there was conversation at the end or during the session. A few participants said that beyond the use of art materials, the therapist sometimes engaged them in interventions such as guided imagery, board games, cards, or other techniques, if they wanted to do so.

According to most participants, during therapy they turned off their cell phone, and described this as a decisive factor in therapy that contributed to their ability to disconnect from their daily routine and devote themselves to therapy: “When you come here you unplug yourself from technology for a moment, so you don’t think about the phone, or about the problems waiting for you… or some annoying messages someone is sending you.”

### Parents’ Involvement in Therapy

A few participants noted that they sometimes told their parents about certain things taking place in therapy, but most participants said that their parents know they are in art therapy at school, but did not perceive them as involved: “My parents know I’m here, they signed the consent form and everything, but they don’t know what I’m doing here in detail.”

### Components Facilitating Art Therapy in School

Five components of therapy were experienced by the participants as significant and crucial to therapy and were mentioned as facilitating elements in art therapy in school. The first four are presented in this core idea. The fifth was the relationship with the art therapist, which is discussed separately below.

#### Art

Most participants noted that art is an important component of art therapy sessions and helps them to express themselves to the therapist. They emphasized the ways in which artwork enables release and venting: “It’s an hour when you do something in art, say painting, sewing, knitting… and this is how you show your feelings to the person you are with.”

#### The Opportunity to Share and to Feel Accepted

All the participants noted that there were verbal exchanges between them and the therapist during therapy and described sharing as a significant component of the session. They emphasized the sense of acceptance they experienced: “I come in and then we talk, like all kinds of conversations, I tell her all kinds of things, she tells me… someone to talk to.”

#### A Sense of Calm

Most of the participants mentioned the feeling of calmness they experienced during the sessions as a component that helped them devote themselves to the therapy and experience it as a time in and of itself within the rest of the school day: “Being calm is very significant here.”

#### A Place of My Own

Some participants said that they felt art therapy allowed them to have “a place of their own” at school and described this feeling as a facilitating element: “You feel like this is your place. As soon as I walk in and close the door, I always tell her ‘wow this is great, a little quiet time.’ That’s the way it is.”

### The Relationship With the Art Therapist

#### Client Expectations From the Art Therapist

Some participants noted that at the beginning of therapy, they found it hard to trust the art therapist. A few of them said that they expected to be disappointed by the new relationship: “To be honest, at first I thought she would be against me. Like, I came and said well, no way, I don’t know her, she doesn’t know me, what can come out of it?”

Some participants noted that during art therapy, as the relationship grew, they began to develop expectations from the therapist, based on how they experienced her, such as calmness, respect, acceptance, and being a therapeutic figure within the educational staff: “I expect her not to be a teacher, not to behave like a teacher… she gives us some calm, so I expect her to do that in the rest of the session.”

#### The Relationship With the Art Therapist and the Therapeutic Alliance

All the participants described the relationship with the art therapist as good and open, where they could share and say everything and feel comfortable: “A good relationship, I talk with her about almost everything.” Some participants described the therapist as a significant figure in their lives at school, and her ability to listen to them and be interested in them as a facilitating factor in the therapeutic process. Some of the participants described their experience with their therapist as similar to having a mother: “She’s like… I I’m debating myself whether to say it, she’s like a mother.”

Most participants described the relationship as characterized by feelings of trust and confidence, confidentiality, caring, respectfulness, and mutuality: “She also tells me that she feels totally comfortable with me, and so do I, and we can talk.”

#### Encounters Outside the Therapy Room

All the participants mentioned that they occasionally saw the art therapist around the school, outside the therapy room, but did not experience this as a problem and in some cases even the opposite. Most participants described the out-of-the-room encounters as shorter and less personal than the encounter during the therapeutic session. Some participants noted that the presence of the art therapist at school contributed to their sense of security at school simply by knowing that she is there and available to them, as well as when dealing with difficulties that arise as part of the school routine, both socially and educationally: “She saw me and told me ‘come with me’, so I went, I calmed down and went to work here.”

### Impact of Therapy on the StudentsDealing With Daily Difficulties

All the participants described art therapy as contributing to their ability to cope with a range of difficulties in their lives such as dealing with fears, reducing stress, and managing their load of coursework and other areas such as anger control, changing behaviors and habits, and contributing to daily conduct: “Let’s say controlling my anger. Like I’m full of energy and that, but like, keeping it down. There are times when you need to be serious and I’m not a serious person, so it helps me be a little bit more serious.” A few participants emphasized dealing with episodes of ostracism at school: “I was ostracized twice before… so my trust in people went down, and down and down, so now it’s like this suddenly, like, it’s going to be a kind of renewal.”

### Sharing and Venting Allows for Relief

Most participants described venting in art therapy as a way to reduce their emotional burden and continue the rest of the day and week within a better mood and less of a sense of loneliness. They stated that being able to share eased their sense of difficulty, and helped them immediately after sharing in the session, and during the week when they felt problems or pain, by knowing that they would soon be able to share and relieve themselves of this burden: “It really gives you a good feeling… if I tell her something I don’t tell others, and it’s fun because it takes a weight off of you.”

### A Source of Support Within the School

Most participants described art therapy as a significant source of support for them at school, and their lives in general. They stated that availability in itself of a school therapeutic service not only allows for a unique experience of support within the school but also sometimes enables them to experience the whole school as a place that also provides emotional support for academic and social problems: “It’s the greatest source of support you can give a person who is going through a social or educational crisis.”

### Enhancement of Self-Confidence and Social Relationships

Some participants noted that as a result of therapy, they felt more self-confident, and some participants described a stronger social relationships, and emphasized communication and self-expression, along with a feeling of increased emotional resilience: “I’m not afraid to be hurt, I feel I can say whatever I want without hurting people, but I also need to talk. I have to talk; I have to express myself.”

Some participants make the point that it is important to be treated during adolescence, when boys and girls face many challenges such as low self-confidence, difficulties in self-acceptance, identity formation and self-determination, and dealing with stigma: “This is so important at my age, particularly when so many people think ‘I’m fat I’m ugly’… It gives me the feeling I can be whatever I want, and I shouldn’t feel bad about myself and all that stuff.”

### Feeling Better Later in the Day and When Back in Class

Some participants indicated that art therapy helped them relax later during the day at school, or at home, after the session, and that the session positively affected their feelings and experiences later in the day, and even contributed to their work in class after the session: “Suddenly there is higher energy and it is fun, it gives me more drive, and ‘let’s study and learn’.”

### Additional Art Therapy Hours Might Increase the Impact of Therapy

Most participants noted that they were satisfied with the art therapy they received at school and did not see any need to change it. When asked what could have been more helpful, some participants said that more art therapy hours for the same client could increase the effectiveness of therapy: “I think this specific therapy should be given to students, let’s say, more hours for students who are having emotional difficulties… more hours for students.”

## Discussion

This study examined adolescents’ perceptions of art therapy in the school setting, thus contributing to previous research on art therapy in the education system from the perspective of therapists, counselors, educators, administrators, and supervisors (Regev et al., 2015; Snir et al., 2018), as well as previous studies that have examined the perceptions of elementary-school children in art therapy at school (Deboys et al., 2017; McDonald et al., 2019a). The findings were organized into five domains that emerged from the interviews: the first domain dealt with referrals and initial engagement with therapy, the second with the setting within school, the third with the nature of therapy at school, the fourth with the relationship with the art therapist, and the fifth with the impact of art therapy on the clients.

The findings for the first domain suggest that the art therapy was at times poorly defined initially to the clients, or that they themselves could not characterize it adequately, which led to a lack of involvement at the start. This is similar to the perceptions of elementary school children, some of whom had problems clearly expressing the goals of therapy and the reasons for their referral to art therapy (Deboys et al., 2017).

The findings suggest that the therapy tended to be presented differently to each client or was perceived as such. Being referred to psychotherapy by an authority, such as parents or teachers, may lead to uncertainty about the therapy process and skepticism regarding its outcomes (Everall and Paulson, 2002; Gibson et al., 2016). It is possible that the staff members who suggested art therapy to the students attempted to adjust their description to the student as a function of personality or type of problem to convince them to agree to art therapy and view therapy in a positive light. In other cases, the staff members may not have known enough about art therapy or how to present it to the students. Although the participants indicated that art therapy was offered to them voluntarily and they were not forced to take part, a referral by an authority figure within the school, rather than a personal process leading to therapy, could have led to a poorer understanding, doubts and sometimes a total lack of knowledge of what art therapy entails. This meant that some students entered into art therapy without clear expectations, and had problems formulating the goals of therapy. However, this initial reaction may have also prompted certain students to give therapy “a chance.” The analysis of the interviews suggested that some participants initially agreed to art therapy because it got them out of class and let them have fun instead. All the participants nevertheless stated that after a period of time of art therapy, they realized that they were engaged in a personal and emotional process focusing on them and allowing them to express their feelings without fear of judgment.

In terms of the therapeutic setting within the school, participants indicated that the equipment and materials were satisfactory and did not experience holidays or school vacations as disruptive to the therapeutic process. This contrasts with previous findings on the part of therapists who generally feel that the ongoing therapeutic flow is interrupted by the many holidays (Snir et al., 2018; Danieli et al., 2019). The students’ sense of continuity may be related to their perception of art therapy as part of the curriculum with its holiday schedule, or their position as clients who are grateful for the therapeutic time they receive and are not aware that other alternatives exist. This again contrasts with the point of view of therapists who have the professional background and are fully aware of the potential benefits of regular sessions. Another discrepancy between therapists and clients emerged in clients’ perceptions of confidentiality. The findings showed that the participants, mainly those in regular education settings felt confidentially were preserved as regard both teachers and other students. This finding is consistent with the claim that psychotherapy within the school setting contributes to lessening the stigma of therapy (Snir et al., 2018), because it allows clients to feel comfortable about leaving the classroom for therapy, and sometimes gives them a sense of being special, or pride about being chosen to have therapy rather than sit in class.

The findings suggested that school-based art therapy has specific characteristics. For example, having a therapeutic hour during a stressful school day gives the students an opportunity to relax, and the art therapy room is perceived as a shelter. In addition, when the therapist is perceived as a supportive figure, the whole school experience may be perceived as supportive or allows for greater acceptance.

In addition to art, the feeling of acceptance, and the opportunity for verbal sharing that were found to be facilitating components, the sense of calm and the therapy room as a “place of my own” were dominant elements in the participants’ experience of art therapy in school. Although recent approaches have promoted community-based models for art therapy (Kapitan et al., 2011; Spong and Waters, 2015), some clients still apparently feel the need for the individual classic approach in the art therapy room, and the dynamic approach, which the participants in this study perceived as appropriate for them.

Students at times used the word “mother” to describe their relationship with the art therapist. In his many essays, Winnicott described the therapist’s role in a similar way to that of the mother who provides a “holding environment,” which enables the self-development of the child, and the analytic process for the client (Winnicott, 1963). The students’ experience of being accepted and not subject to judgment during therapy, along with their sense of respect and mutuality, appear to have contributed to the strength of the therapeutic alliance and assisted the therapeutic process. This finding is consistent with previous interviews of adolescents which showed that these are seen as essential features in developing trust with the therapist (Eyrich-Garg, 2008; Gibson et al., 2016).

The participants described the presence of the art therapist at school as making them feel safer and helping them deal with daily difficulties. This finding is congruent with data showing that therapeutic staff at school increases the students’ sense of protection (Mann et al., 2019).

The participants described art therapy as fostering calm and enabling them to continue their day at school feeling better and concentrating more. These finding’s echo remarks made by primary school children (Deboys et al., 2017; McDonald et al., 2019a). In addition, art therapy has been shown to contribute to dealing with self-confidence issues and social relationships. The clients pointed out that therapy was important at adolescence which is a challenging period of life (Dowdy et al., 2015). The interviews showed that social issues and identity formation were significant concerns for these adolescents, and were reflected in their perceptions of art therapy, its goals, and contributions. These findings are consistent with Erikson’s psychosocial theory that defines adolescence as a stage of identity vs. role confusion and emphasizes the social reality of the person (Mitchell and Black, 1995).

Since most of the participants had never taken part in psychotherapy outside of school, they were unfamiliar with both art-therapy but also the therapeutic process in general. This might explicate the participants’ inability to describe the negative or restrictive factors of school art therapy. It might also be due to the students’ age, or to their point of view as clients, as was the case for their positive and uncritical evaluations of the art therapy room and equipment.

## Limitations and Future Research

Overall, the findings indicated that the participants experienced art therapy in school as a positive and beneficial element in their lives. One of the criteria for recruiting participants was that at the time of the interview, the clients had to be in at least their 2nd year of art therapy in school. This was done to ensure a broader perspective. Thus, the students who took part in this study may already have developed a positive point of view about art therapy. Future research should examine the perceptions of students who dropped out of art therapy which could shed light on the difficulties and those areas that should be optimized to improve in art therapy in school. In addition, when examining the findings, we did not find any compelling evidence that would suggest that there was a difference in perceptions in terms of the age and/or the gender of the participants. Future studies should test for potential differences in perceptions between the participants’ gender and age with larger samples.

Another criterion for recruitment was high verbal ability. However, students with lower verbal abilities may experience art therapy in different ways. Future work should implement other assessment techniques than interviews such as reflective client diaries, questionnaires, or art materials and expressive tools that can provide valuable non-verbal data.

Finally, this was a qualitative study on a small sample that reported the subjective point of view of the participants. Future research should examine the central themes that emerged from this study, and their frequency in a larger sample. Studies should also examine changes in perceptions during the therapeutic process, and the effectiveness of art therapy in schools through questionnaires, observations, and a control group.

## Data Availability Statement

The datasets generated for this study are available on request to the corresponding author.

## Ethics Statement

The studies involving human participants were reviewed and approved by Ethics Committee of the Faculty of Health and Welfare at the University of Haifa, and by Chief Scientist at the Ministry of Education, Israel. Written informed consent to participate in this study was provided by the participants’ legal guardian/next of kin.

## Author Contributions

SH, DR, and SS: study design, writing, and data analysis. SH: conducting research. RR-K: assist in the writing process. All authors contributed to the article and approved the submitted version.

### Conflict of Interest

The authors declare that the research was conducted in the absence of any commercial or financial relationships that could be construed as a potential conflict of interest.
